# Symmetrical Drug-Related Intertriginous and Flexural Exanthema (SDRIFE) Unfolded: Diagnostic Pitfalls and Psoriatic Confounders Among Flexural Dermatoses

**DOI:** 10.7759/cureus.93524

**Published:** 2025-09-29

**Authors:** Gautam K Singh, Suyog S Dhamale, Amit Jain, Anshu Baghel, Vidyadhar R Sardesai

**Affiliations:** 1 Department of Dermatology, Bharati Vidyapeeth Medical College and Bharati Hospital, Pune, IND

**Keywords:** diagnostic challenge, drug eruption, flexural dermatoses, inverse psoriasis, symmetrical drug related intertriginous and flexural exanthema (sdrife)

## Abstract

Symmetrical drug-related intertriginous and flexural exanthema (SDRIFE) is an uncommon, symmetrical drug rash that impacts intertriginous areas without exhibiting systemic symptoms. Its occurrence in a patient with pre-existing flexural dermatoses such as inverse psoriasis can complicate diagnosis, as in our case, where the eruption was initially suspected to represent a psoriatic flare. We present a 53-year-old female with biopsy-proven inverse psoriasis who experienced a broad symmetrical erythematous rash in flexural areas after receiving amoxicillin-clavulanate treatment. Clinical assessment, histopathological results, and Naranjo scoring supported a diagnosis of SDRIFE. The case emphasizes diagnostic challenges arising from shared characteristics with conditions like candidiasis, acute generalised exanthematous pustulosis (AGEP), and inverse psoriasis. SDRIFE was preferred over the flare of inverse psoriasis due to lesion appearance, temporal correlation with drug exposure, and absence of systemic symptoms. Interestingly, previous flexural psoriasis might have contributed as a risk factor via barrier impairment or recall phenomenon. This case emphasizes the importance of increased clinical awareness and histological assessment in flexural lesions, particularly in individuals with pre-existing dermatoses.

## Introduction

Flexural dermatoses are a heterogeneous group of skin conditions that are characterised by involvement of intertriginous areas such as the axillae, groin, inframammary folds, perineum, and abdominal creases. Common conditions affecting flexural areas include allergic and irritant contact dermatitis, which often result from prolonged exposure to sweat, topical agents, or friction from clothing; inverse (flexural) psoriasis; candidal intertrigo; seborrheic dermatitis; tinea cruris; erythrasma; and drug-induced eruptions like symmetrical drug-related intertriginous and flexural exanthema (SDRIFE) [1]. Flexural areas of the body are uniquely vulnerable to combination of moisture, friction, warmth, and limited airflow, conditions that can compromise the skin barrier and predispose individuals to specific dermatological disorders [2]. Although these flexural dermatoses often share overlapping clinical signs such as erythema, maceration, irritation, and general discomfort, their root causes can differ markedly. They may stem from autoimmune processes, inflammatory conditions, infections, or even adverse drug reactions [3]. At times, these dermatoses may coexist with one another or arise in the setting of an already existing dermatological condition, further complicating the clinical picture [4]. This convergence of symptoms often complicates the diagnostic process, particularly in flexural areas where hallmark features like scaling or satellite lesions may be diminished or absent due to persistent occlusion and moisture [1]. Accurate diagnosis is critical, as management strategies vary considerably depending on the underlying cause. Against this backdrop, we present a rare case of SDRIFE in a patient with pre-existing inverse psoriasis, highlighting the diagnostic pitfalls and clinical implications of overlapping flexural dermatoses.

## Case presentation

**Figure 1:**
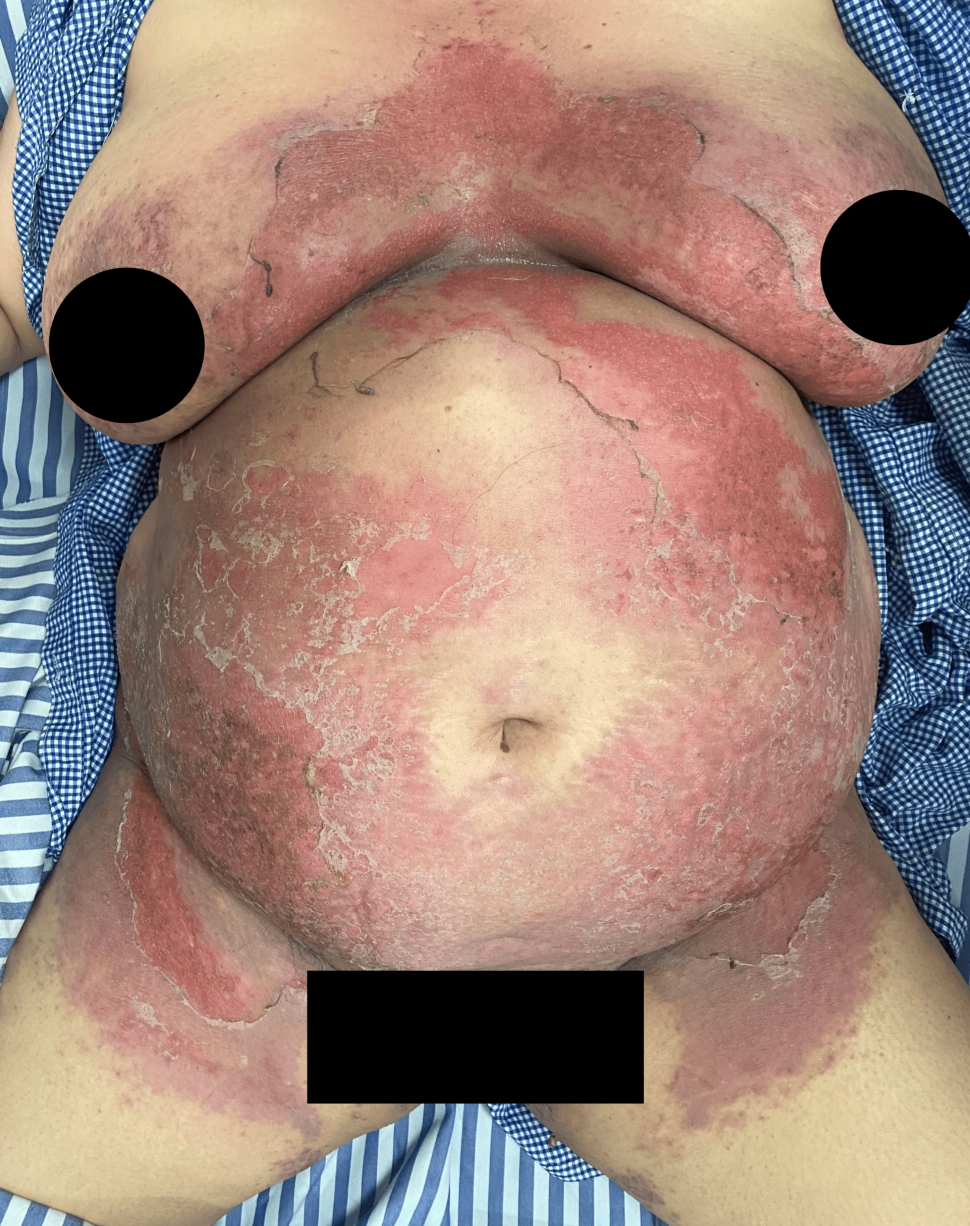
Presence of diffuse erythematous rash over bilateral inframammary area, bilateral genitocrural folds, and abdomen.

A 53-year-old obese female presented to the dermatology outpatient department of a tertiary care centre with complaints of a burning, erythematous rash predominantly affecting the flexural regions for the past seven to 12 days. The eruption initially appeared over the perineum and gluteal cleft, later spreading to involve the groin, inframammary folds, axillae, and the area beneath the abdominal pannus (Figure 1).

The rash began as a mildly symptomatic erythematous exanthema and gradually evolved into extensive, symmetrical, non-palpable erythema with superficial exfoliation, associated with pruritus and burning pain over the last seven to 10 days. There was no history of fever, chills, myalgia, arthralgia, and sore throat, and she denied any recent vaccination or preceding upper respiratory or gastrointestinal illness. There was also no history of topical application of any new cosmetic, medicated, or irritant substance, reducing the likelihood of contact dermatitis.

The patient was a known case of biopsy-proven flexural psoriasis for the past three to four years, with a history of multiple relapses and remissions under regular dermatological follow-up. At the time of evaluation, she had not been on any active treatment for the previous two to three months and was only applying emollients. Her medical history included poorly controlled type 2 diabetes mellitus and hypertension, both diagnosed approximately seven to eight months earlier, for which she was on oral hypoglycaemic and antihypertensive agents including metformin 500 mg, telmisartan 40 mg, amlodipine 5 mg, and cilnidipine 10 mg. She also had a history of cerebrovascular accident (CVA) two years prior and was receiving antiplatelet therapy with aspirin 75 mg and clopidogrel 75 mg.

Approximately 10-14 days prior to presentation, she developed a painful, erythematous, raised lesion over the abdomen, diagnosed clinically as a furuncle, for which she was prescribed oral Amoxicillin + Clavulanic Acid 625 mg twice daily. Although she had received the same antibiotic in the past without adverse events, this episode was followed by the onset of a widespread flexural rash within three to four days of initiating the medication, raising suspicion for a drug-induced aetiology. The antibiotic was chosen empirically as first-line therapy for a bacterial furuncle, and at initiation, no link was made with the patient’s psoriasis history because she had tolerated the same drug in the past without incident. However, the subsequent onset of a symmetrical flexural eruption within days of drug exposure led us suspect Amoxicillin + Clavulanic Acid as the likely trigger, distinguishing the reaction from a psoriatic flare.

**Figure 2:**
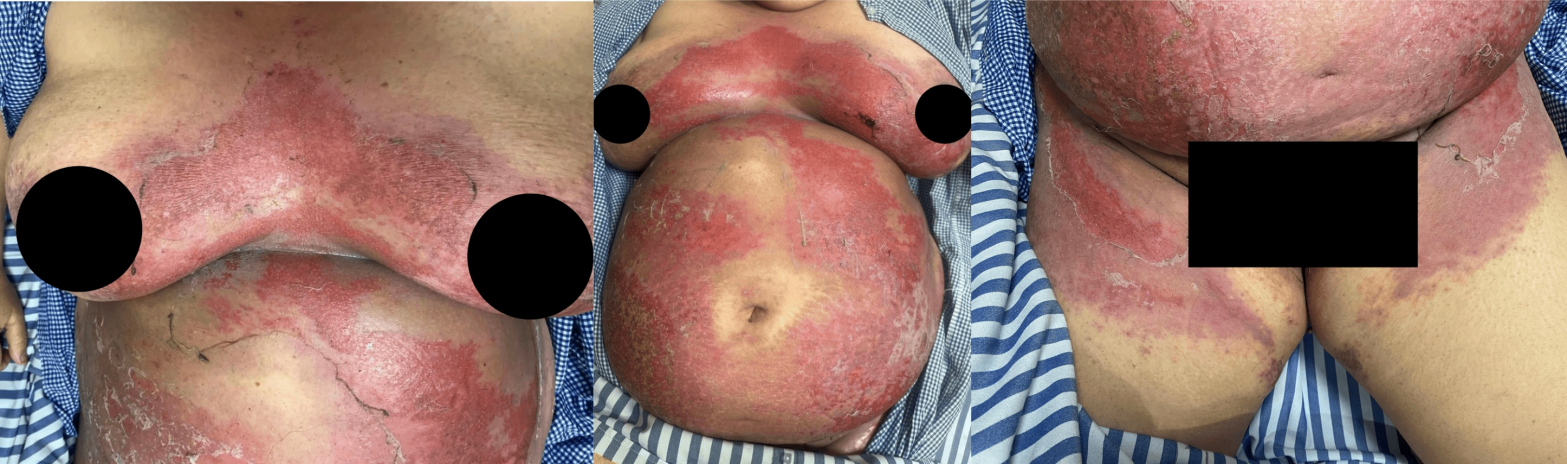
Presence of well-defined, symmetrical erythema accompanied by superficial desquamation involving the groin, axillae, inframammary areas, and lower abdomen.

In her previous flares, inverse psoriasis had typically manifested as well-demarcated erythematous patches within axillae, groin, and inframammary folds, without appreciable scaling owing to constant flexural moisture. In contrast, the current eruption showed well-defined, symmetrical erythema accompanied by superficial desquamation involving the groin, axillae, inframammary areas, and lower abdomen (Figure 2).

**Figure 3:**
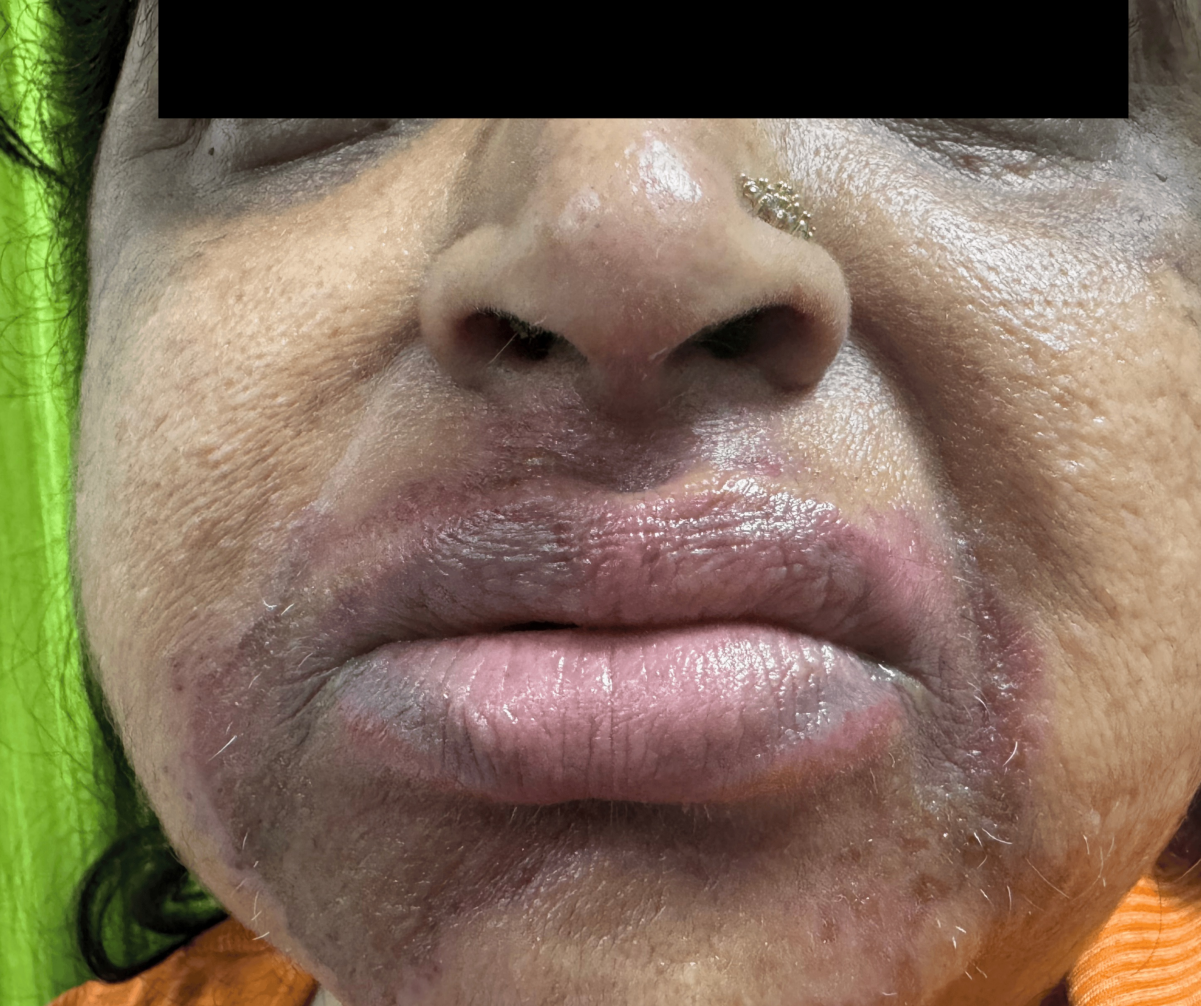
Presence of well-defined erythema with superficial desquamation over perioral area.

There were no pustules, plaques, or scaling typically seen in active psoriatic lesions. Extension to non-classical site, such as perioral involvement, was present (Figure 3).

**Figure 4:**
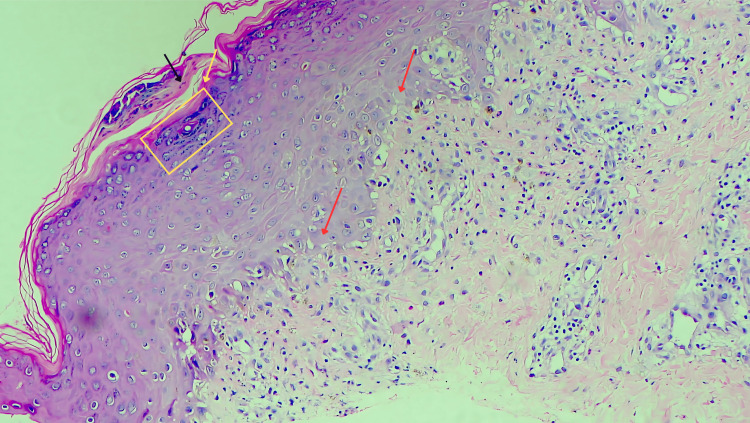
Haematoxylin and eosin (H&E) staining at scanner magnification (10x) showing parakeratosis (black arrow) and hypergranulosis (yellow box) in the epidermis. Few apoptotic keratinocytes (red arrow) are seen high up in the epidermis.

**Figure 5:**
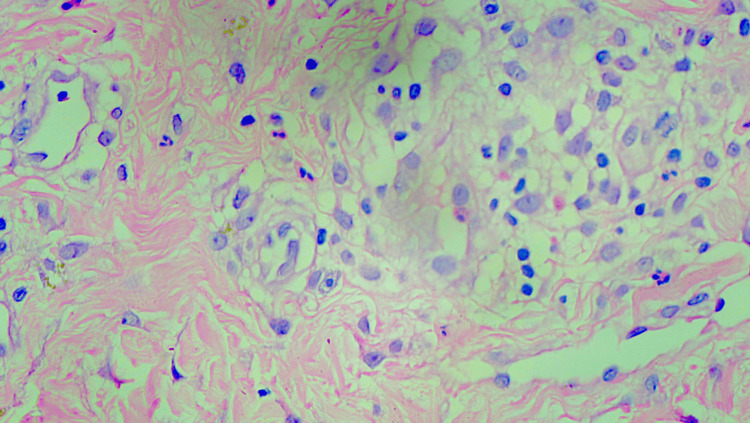
Haematoxylin and eosin (H&E) at higher magnification (100x) demonstrated the interphase dermatitis in the form of vacuolar change along with infiltration of neutrophils and eosinophils.

This deviation from her usual psoriatic morphology made a psoriatic flare less likely and favoured a drug-induced exanthem. There was no facial involvement, mucosal involvement, acral desquamation, or targetoid lesions. She was admitted for further evaluation with a provisional differential diagnosis that included a flare of flexural psoriasis, acute generalised exanthematous pustulosis (AGEP), and symmetrical drug-related intertriginous and flexural exanthema (SDRIFE). Laboratory investigations revealed neutrophilic leucocytosis, but all other haematological and biochemical parameters, including liver and kidney function tests, remained within normal ranges. Absolute eosinophil count was not raised, thereby reducing the likelihood of AGEP. A potassium hydroxide (KOH) mount from the affected area was negative for fungal elements. A skin biopsy was performed, given the atypical presentation and temporal relationship with drug intake. Histopathology revealed parakeratosis, hypergranulosis, and scattered apoptotic keratinocytes in the upper epidermis, along with vacuolar interface dermatitis (Figure 4) and a mixed neutrophilic-eosinophilic infiltrate in the papillary dermis, findings consistent with SDRIFE (Figure 5).

To further clarify the distinction, Table 1 summarizes the key histopathological differences between SDRIFE, psoriasis, and AGEP, supporting the diagnostic interpretation in this case. Considering the clinical features, histopathological findings, and the Naranjo Adverse Drug Reaction Probability Scale score [8], yielded a score of 6, consistent with a probable adverse drug reaction, a diagnosis of SDRIFE was made.

**Table 1:** Key histopathological features distinguishing SDRIFE, psoriasis, and AGEP. Adapted from: [3,5-7]. SDRIFE: symmetrical drug-related intertriginous and flexural exanthema, AGEP: acute generalised exanthematous pustulosis.

Feature	SDRIFE	Inverse Psoriasis	AGEP
Epidermal changes	Parakeratosis, vacuolar interface, apoptotic keratinocytes	Regular acanthosis, elongation of rete ridges, suprapapillary thinning	Spongiform subcorneal pustules, spongiosis
Granular layer	Hypergranulosis	Often diminished/absent	Variable, usually preserved
Dermal infiltrate	Mixed eosinophilic and neutrophilic	Predominantly neutrophilic, with dilated vessels	Dense neutrophilic with papillary dermal oedema

In view of the suspected drug-induced reaction and the underlying risk of triggering pustular psoriasis, the patient was started on oral cyclosporine at a dose of 100 mg three times a day. The skin lesions responded well, showing marked improvement with decreased erythema and gradual re-epithelialization over the subsequent days.

## Discussion

Symmetrical drug-related intertriginous and flexural exanthema (SDRIFE) is an uncommon, often missed cutaneous adverse reaction to drugs, defined by a symmetrical red rash affecting the perineum and flexural zones after systemic drug exposure, with no systemic symptoms present and other flexural dermatoses ruled out. SDRIFE has been noted in connection with various medications, encompassing frequently prescribed drugs like allopurinol, antihypertensives, heparin, and non-steroidal anti-inflammatory medications, along with chemotherapy agents, monoclonal antibodies, and iodine-containing contrast substances. Among these, antibiotics, especially β-lactams like amoxicillin, continue to be the most commonly identified agents [1,2].

In a few reported cases, SDRIFE has been observed in areas previously affected by other flexural dermatoses, triggered by repeated exposure to a medication that was initially well tolerated [1]. We found it pertinent to report this case of SDRIFE, wherein the eruption manifested over pre-existing psoriatic areas following re-exposure to the same drug possibly because of latent sensitization, where the first exposure primes T-cells without overt clinical reaction, but subsequent exposures elicit a delayed-type hypersensitivity response. This case highlights the phenomenon of SDRIFE localising to previously involved dermatoses, suggesting a possible site-specific recall response and underscoring the importance of considering this entity in the differential diagnosis of recurrent flexural eruptions.

A set of clinical diagnostic criteria has been proposed for SDRIFE, which includes: 1. exposure to a systemically administered drug, either as a first or repeat dose; 2. sharply demarcated erythema of the gluteal/perineal and/or inguinal regions; 3. involvement of at least one additional flexural or intertriginous site; 4. symmetrical distribution; and 5. absence of systemic symptoms and signs [4]. The case described here met all of these criteria, supporting the final diagnosis of SDRIFE.

The diagnostic process in this case was difficult due to the overlap among various flexural dermatoses. The symmetrical distribution of the rash over areas previously affected with flexural psoriasis caused significant confusion; however, absence of systemic abnormalities on investigation, corroborative histopathological findings, and a well-defined temporal association with drug administration collectively supported a diagnosis of symmetrical drug-related intertriginous and flexural exanthema (SDRIFE). Nonetheless, the diagnostic clarity is often compromised in flexural regions, where moisture, occlusion, and friction may obscure characteristic clinical features. For example, chronic flexural psoriasis compromises the skin barrier, thereby increasing susceptibility to secondary infections such as candidiasis or intertrigo. Candidiasis commonly affects major skin folds and may present as erythematous papules and pustules, whereas erythrasma, caused by *Corynebacterium minutissimum*, typically appears as reddish-brown macules and patches in the axillary or inguinal regions and demonstrates coral-red fluorescence under Wood’s lamp examination. Beyond the diagnostic intricacies, managing the condition posed its own set of challenges and therapeutic decision making was equally complex. The use of immunosuppressants such as cyclosporine in a patient of hypertension required monitoring and carried the risk of worsening undetected infections like candidiasis, while withholding them could allow unchecked progression of a drug-triggered inflammatory dermatosis. Similarly, empirical treatment of presumed intertriginous infections such as antibiotic therapy for suspected intertrigo may inadvertently precipitate drug-induced eruptions or promote fungal overgrowth, thereby further complicating the clinical picture [9].

Among the differential diagnoses, acute generalised exanthematous pustulosis (AGEP) warranted particular consideration. Although AGEP can initially localize to flexures, it is typically marked by an abrupt onset of numerous, non-follicular pustules on an erythematous base, followed by widespread involvement. AGEP is frequently accompanied by systemic features such as fever, leucocytosis, facial or acral oedema, and, occasionally, targetoid or purpuric lesions and erythroderma. β-lactam and macrolide antibiotics, as well as calcium channel blockers, are well-established triggers. In contrast, SDRIFE rarely features vesicles or pustules, and when present, these are neither as numerous nor as confluent as in AGEP. Importantly, SDRIFE lacks the systemic toxicity and extensive cutaneous involvement that characterizes AGEP, favouring its diagnosis in this patient [9].

To establish an accurate diagnosis, it was essential to distinguish SDRIFE from other flexural dermatoses, including inverse psoriasis, Hailey-Hailey disease, and pemphigus vegetans. Hailey-Hailey disease is often characterised by fragile blisters and erosions in body folds, most notably in the axillae and groin, which can evolve into malodorous, vegetative lesions with painful fissuring. Similarly, pemphigus vegetans typically presents with thickened, vegetating plaques in intertriginous areas and is frequently associated with mucosal involvement. These clinical features, however, are not observed in SDRIFE, which assists in differentiating it from these other conditions during evaluation [9].

Given her established diagnosis of biopsy-confirmed inverse psoriasis, it was important to consider whether the current eruption represented a flare of psoriasis, a coexisting episode of SDRIFE, or whether the presence of inverse psoriasis may have predisposed her to SDRIFE. Notably, psoriatic lesions have been identified as potential sites for delayed-type hypersensitivity reactions [4]. Although SDRIFE pathogenesis remains incompletely understood, it is hypothesised to involve a type IV hypersensitivity reaction, particularly subtypes IVa (T helper 1 (Th1) and macrophage-mediated) and IVc (CD8+ T-cell-mediated) [2]. These mechanisms, however, do not fully account for all presentations of SDRIFE, suggesting a multifactorial basis.

One of the more recently proposed models is the pharmacologic interaction with immune receptors (p-i) concept, which suggests that drugs may directly interact with T-cell receptors without prior antigen processing or major histocompatibility complex (MHC) presentation. In the context of psoriasis, which is driven by sustained T-cell activation and a Th1/Th17-skewed immune response, the p-i model offers a plausible explanation for exaggerated reactions. The abundance of pre-activated T cells in psoriatic skin may have lowered the threshold for aberrant drug T-cell receptor (TCR) interactions, allowing Amoxicillin + Clavulanic Acid to directly trigger an effector response upon re-exposure. This could explain why SDRIFE may develop following initial drug exposure or in response to agents previously considered non-immunogenic. Another proposed mechanism is the recall phenomenon, wherein drug- or allergen-induced eruptions preferentially localize to anatomical sites previously affected by unrelated dermatoses such as psoriasis, diaper dermatitis, or intertrigo. In this case, SDRIFE lesions occurred over areas previously affected by inverse psoriasis, suggesting a possible recall response as well as the potential for clinical overlap with pre-existing flexural dermatoses. Inverse psoriasis itself was ruled out as the primary cause based on the temporal correlation with amoxicillin use and characteristic histopathologic findings [1].

Further mechanistic insights into SDRIFE have been provided by a study conducted by Chaudet et al. which revealed a markedly higher proportion of Th17 cells and an elevated Th17:Th2 ratio in SDRIFE cases compared to eczema and psoriasis [5]. These findings support the notion that SDRIFE is predominantly mediated by Th1 and potentially Th17 immune pathways [5]. This immunological profile not only aids in distinguishing SDRIFE from other inflammatory dermatoses but also highlights the potential for developing targeted therapeutic strategies based on these pathways. Cyclosporine was chosen to rapidly control drug-induced inflammation while avoiding systemic corticosteroids, which carry the risk of precipitating pustular psoriasis. The patient was closely monitored for blood pressure and renal function during therapy.

## Conclusions

This case illustrates the diagnostic complexity of SDRIFE coexisting/manifesting over the previously affected areas of flexural psoriasis, raising the possibility that barrier impairment or recall phenomenon in psoriatic skin may predispose to SDRIFE, warranting further study. Moreover, the occurrence of the disease in a patient where it has been used safely before raises further queries and can provide important insights into the disease pathophysiology. Treatment decisions must balance the risks of immunosuppression against the progression of drug-induced inflammation. This case reinforces the need for a meticulous drug history as pharmacotherapy continues to evolve, and drug-induced reactions become increasingly diverse. Heightened awareness of SDRIFE and its clinical mimics is essential for timely and effective management. Histopathological confirmation should be sought when the morphology is atypical or overlapping, as it provides critical differentiation. By maintaining a high index of suspicion and integrating clinical, histological, and temporal parameters, clinicians can improve diagnostic precision and avoid unnecessary interventions in diagnostically ambiguous flexural presentations; also, interconnections in the aetiopathogenesis of different inflammatory flexural dermatoses may be worth exploring.
